# Effect of trimetazidine on preventing contrast-induced nephropathy in diabetic patients with renal insufficiency

**DOI:** 10.18632/oncotarget.19519

**Published:** 2017-07-24

**Authors:** Ziliang Ye, Haili Lu, Qiang Su, Xinhua Xian, Lang Li

**Affiliations:** ^1^ Department of Cardiology, The First Affiliated Hospital of Guangxi Medical University, Guangxi Cardiovascular Institue, Nanning, Guangxi, China; ^2^ Guangxi Medical University, Nanning, Guangxi, China

**Keywords:** trimetazidine, contrast-induced nephropathy, diabetic patients, renal insufficiency

## Abstract

**Background:**

Our study sought to assess the effect of trimetazidine (TMZ) on preventing contrast-induced nephropathy (CIN) in diabetic patients with renal insufficiency.

**Materials and Methods:**

106 diabetic patients with renal insufficiency who were undergoing coronary angiography (CAG) and/or percutaneous coronary intervention (PCI) were enrolled in this study. Standard hydration was administered to both groups (the TMZ group and the control group). In the TMZ group, patients were orally administered TMZ for 48 hours before and 24 hours after CAG and/or PCI. Serum creatinine (Scr), cystatin C and the glomerular filtration rate (eGFR) were measured before as well as 24 hours, 48 hours and 72 hours after contrast media injection. The incidence of CIN and major cardiovascular events (MACE) was also evaluated in both groups.

**Results:**

Scr, cystatin C and the eGRF in the TMZ group were better than those in the control group after 24 hours (OR: 0.78, 95% CI: 0.54–0.82; OR: 0.66, 95% CI: 0.62–0.73; OR: 1.2, 95% CI: 1.02–1.53, respectively), 48 hours (OR: 0.69, 95% CI: 0.52–0.73; OR: 0.76, 95% CI: 0.69–0.84; OR: 1.5, 95% CI: 1.25–1.68, respectively) and 72 hours (OR: 0.82, 95% CI: 0.77–0.91; OR: 0.85, 95% CI: 0.71–0.92; OR: 1.67, 95% CI: 1.33–1.72, respectively). The incidence of CIN (9.26% vs 16.67%) and MACE (7.41% vs 18.51%) in the TMZ group was significantly lower than that in the control group (*P* < 0.05).

**Conclusions:**

Our study suggests that TMZ could reduce the incidence of CIN and MACE in diabetic patients with renal insufficiency who are undergoing CAG and/or PCI.

## INTRODUCTION

At present, with the development of diagnostic technology and interventional therapy, contrast medium is widely used during radiation intervention, especially in coronary angiography (CAG), and contrast-induced nephropathy (CIN) caused by the contrast agent has attracted clinicians’ attention [1–3]. At present, CIN is defined as the impairment of renal function and determined by either a 25% increase in Scr from baseline or a 0.5 mg/dL increase in the absolute value within 48 to 72 hours following intravenous contrast administration [4–5], but the definition and diagnostic criteria of CIN are not unified.

CIN has a variety of high-risk factors, including renal dysfunction and diabetes [6–8]. Studies have shown that in patients with diabetes, the incidence of CIN is approximately 5.7%–29.4% [9–10]. As is known, diabetes mellitus is a disease that will damage multiple organs and systems, especially the kidney and small vessels. Therefore, patients with diabetes often have renal insufficiency [11]. Once CIN occurs, the incidence rate of cardiovascular and renal adverse events and the short-term and long-term mortality rates will significantly increase, which will seriously impact patient survival [12]. Meanwhile, CIN will cause an increase in medical costs and trouble for doctors and patients. CIN has become a hot spot in kidney, heart, radiology and other research fields, which has attracted the attention of clinicians. However, there is no effective drug for the treatment of CIN, and research on CIN has mainly focused on prevention.

Hydration is one of the most widely used methods for preventing CIN. Patients are given saline solution (1–1.5 mL/kg/hour) six hours before the operation and six hours after the operation, or sodium bicarbonate is intravenously injected once per hour starting one hour before the operation until the end of the operation [13–14]. Except for hydration, there are no other effective methods or drugs that can prevent CIN. In recent years, people have reported a variety of drugs that can be used in the prevention of CIN, such as hemofiltration [15–16], low-dose dopamine [17–18], calcium-channel antagonists [19–20], loop diuretics [21], mannitol [22–23], adenosine [24] and atrial natriuretic peptide [25–26]. However, the conclusions from these studies are still controversial and need to be further confirmed on a larger scale. Therefore, the search to find an effective prevention program for CIN has become very important.

Research results have shown that trimetazidine (TMZ) [27–28] has a protective effect upon the heart by inhibiting oxidative stress, scavenging oxygen free radicals and improving lipid metabolism. Its pharmacological characteristics make it a promising drug for the prevention of CIN. In 1996, Grekas [29] studied the effects of TMZ on renal ischemia reperfusion injury in rats, and the results showed that TMZ could reduce MDA levels, which can reflect the degree of lipid peroxidation, in renal tissue and could effectively prevent oxygen free radical-induced renal injury. Animal experiments conducted by Akgullu [30] have also confirmed that TMZ can effectively reduce the value of MDA levels in the kidney after exposure to contrast agent and increase SOD activity, which reflects free radical scavenging activity.

At present, clinical research has confirmed that TMZ can effectively prevent CIN in patients undergoing CAG and/or PCI. Onbasili [31] carried out a study to compare the effect of conventional hydration combined with TMZ and routine hydration, and the results showed that the incidence of CIN in the TMZ group was significantly lower than that in the conventional hydration group (2.5% VS 16.6%, *P* < 0.05). Despite this, there is still a lack of clinical trials to confirm that TMZ can prevent CIN in diabetic patients with renal insufficiency. Therefore, our study sought to evaluate the clinical effect of TMZ on the prevention of CIN in diabetic patients with renal insufficiency.

## MATERIALS AND METHODS

### Patients

A total of 106 diabetic patients with renal insufficiency who were undergoing CAG or/PCI at our hospital from September 2015 to September 2016 were enrolled in our study. The inclusion criteria were as follows: 1) Age ≥ 18 years old; 2) Diagnosis of diabetes and renal insufficiency (glomerular filtration rate between 30 to 89 mL/min/1.73 m^2^ and renal function stability); 3) Underwent CAG and/or PCI; 4) Provided written informed consent.

The exclusion criteria were as follows: 1) Not undergoing CAG and/or PCI; 2) Acute coronary syndrome; 3) Severe acute heart failure; 4) Left ventricular thrombus; 5) Severe valvular disease that requires surgery; 6) End-stage renal insufficiency, and kidney or heart transplantation; 7) Allergy to contrast agents; 8) Pregnant or lactating women; 9) Malignant tumors; 10) Left ventricular ejection fraction < 35%; and 11) Allergy to TMZ.

### Study protocol

Our research was approved by the Ethics Committee of the First Affiliated Hospital of Guangxi Medical University, and all patients provided written informed consent. All patients were fully informed about the study protocol and signed informed consent forms before the study.

Patients were randomly divided between the TMZ group (*n* = 54) and the control group (*n* = 52) using the random number table method. All patients were administered standard hydration: isotonic saline was given 3 to 12 hours before CAG/PCI and up to 12 hours after the operation at a rate of 1 to 1.5 mL/kg per hour. In the TMZ group, patients were given TMZ (20 mg, three times a day) orally at 48 hours before and 24 hours after CAG and/or PCI. Echocardiographic examination was performed on all patients before coronary angiography.

CAG and/or PCI was performed through the radial or femoral artery. Upon enrollment, coronary angiography and/or PCI was performed according to standard protocols and using standard techniques for CAG and/or PCI. Surgical instruments were selected as recommended by the guidelines recommended and based on the experience of the surgeon; non-ionic osmotic contrast agent (Iodixanol) was used. We did not restrict the use of contrast agents, which was administered as needed.

### Laboratory examinations

Venous blood samples were acquired in the morning before the operation and within 24 hours, 48 hours and 72 hours after CAG, and serum creatinine (Scr) and cystatin (CysC) were tested. The levels of Scr were measured using the colorimetric method [32], and the levels of cystatin C were measured using an immunonephelometric method [33]. The glomerular filtration rate [34] (eGFR) was measured in two ways: eGFR (male) = 186 × (Cr/88.40)−1.154 × age−0.203; and eGFR (female) = 186 × (Cr/88.4)−1.154 × age−0.203 × 0.742 in μmol/l. CIN is defined as the impairment of renal function as determined by either a 25% increase in Scr from baseline or a 0.5 mg/dL increase in the absolute value within 48 to 72 hours after intravenous contrast administration. Major adverse cardiovascular events (MACE) including cardiovascular death, nonfatal myocardial infarction, target vessel revascularization and heart failure.

### Statistical analysis

Results are presented as the number (percent) or the mean ± SD. Categorical variables were compared using the Chi square test. We compared the mean values of continuous variables between the 2 groups by unpaired Student's *t*-test. Differences in the mean values of continuous variables between baseline, and 12 hours, 24 hours and 72 hours after the operation were compared using repeated measures analysis of variance.

In the primary analysis comparing the treatment effects, the baseline-adjusted means and their 95% CIs, as estimated by analysis of covariance (ANCOVA), were compared between the treatments groups (the TMZ group versus the control group). This analysis was carried out while taking into account the variation caused by treatment effects, and age, body height, body weight, high-density lipoprotein, HBA1C, triglyceride, waist circumstance, small dense low density lipoprotein, high molecular weight adiponectin, history of dyslipidemia, history of liver disease, history of cerebrovascular disorders, history of hypertension, history of cerebral hemorrhage, history of transient ischemic attacks, history of cerebrovascular disorders, history of arrhythmia, smoking habit, drinking habit, history of coronary artery bypass grafting, aspirin, clopidogrel sulfate, ACEIs/ARBs, beta-blockers, calcium antagonists, nitrates and GPIIb/IIIa inhibitors were used as covariates. We did not impute missing observations for the primary analysis, but the mixed-effects model for repeated measures was applied as a sensitivity analysis method to examine the effect of missing data. The Secondary analyses were performed in the same manner as the primary analysis. Furthermore, a sensitivity analysis was conducted to evaluate the effect of TMZ between males and females.

All comparisons were planned, and all *p*-values were two-sided. A *p*-value < 0.05 was considered statistically significant. All statistical analyses were performed using SAS (version 9.4 Stata Co. College Station, Texas, USA).

## RESULTS

**Figure 1:**
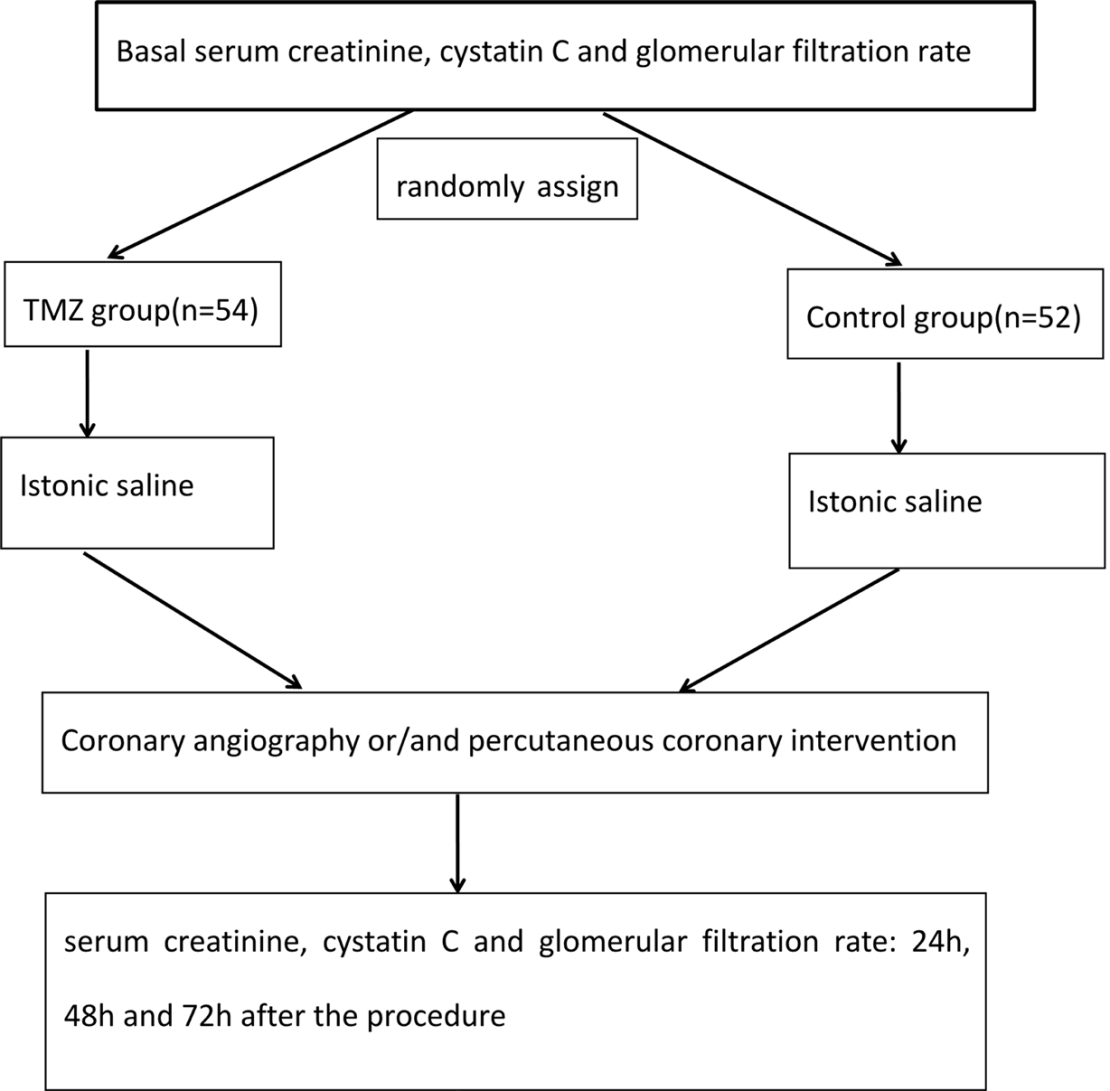
Flow chart of the research study

Laboratory data were not available for 5 patients (the TMZ group: *n* = 3, and the control group: *n* = 2), and 4 patients in the TMZ group withdraw from our study after refusing to use TMZ before the operation. There were two patients in the control group who did not undergo CAG and/or PCI due to other reasons. The 11 patients mentioned above were excluded from our study. Finally, 106 diabetic patients with renal insufficiency were included in our study, and the flowchart of our study is shown in Figure 1.

### Baseline clinical characteristics

Table 1 shows the baseline clinical characteristics of all patients. Of the 106 patients, 63 (59.43%) were male and 43 (40.57%) were female. There was no significant difference in any of the variables besides HBA1C, waist circumstance, history of hypertension, history of arrhythmia, drinking habit and GPIIb/IIIa inhibitors, including age, body height, body weight, high-density lipoprotein, triglyceride, small dense low-density lipoprotein, high molecular weight adiponectin, history of dyslipidemia, history of liver disease, history of cerebrovascular disorders, history of cerebral hemorrhage, history of transient ischemic attacks, history of cerebrovascular disorders, smoking habit, history of coronary artery bypass grafting, aspirin, clopidogrel sulfate, ACEIs/ARBs, beta-blockers, calcium antagonists and nitrates.

**Table 1:** Baseline clinical characteristics

	the TMZ group (*n* = 54)	the control group (*n* = 52)	*P* value
Sex (male)	32 (59.26%)	31 (59.61%)	0.970
Age (year)	63.2 ± 8.3	65.3 ± 8.7	0.2063
Body height (cm)	166.5 ± 6.2	165.3 ± 5.2	0.2837
Body weight (kg)	69.4 ± 11.4	69.9 ± 10.2	0.8126
High density lipoprotein (mg/dL)	47.0 ± 12.3	50.1 ± 14.4	0.2355
HBA1C (%)	6.4 ± 0.6	6.9 ± 0.5	0.0000
Waist circumstances (cm)	86.6 ± 8.2	90.9 ± 9.9	0.0164
Small dense low density lipoprotein (mg/dl)	35.8 ± 12.3	34.2 ± 13.1	0.5181
History of dyslipidemia			0.686
No	26 (48.15%)	23 (44.23%)	
Yes	28 (51.85%)	29 55.77%)	
History of liver disease			0.582
No	36 (66.67%)	32 (61.53%)	
Yes	18 (33.33%)	20 (38.47%)	
History of cerebrovascular disorder			0.346
No	18 (33.33%)	13 (25.00%)	
Yes	36 (66.67%)	39 (75.00%)	
History of hypertension			0.041
No	11 (20.37%)	20 (38.46%)	
Yes	43 (79.63%)	32 (61.54%)	
History of cerebral hemorrhage			0.381
No	21 (38.89%)	16 (30.77%)	
Yes	33 ( 61.11%)	36 ( 69.23%)	
History of transient ischemic attacks			0.065
No	11(20.37%)	19 (36.54%)	
Yes	43 (79.63%)	33 ( 63.46%)	
History of cerebrovascular disorder			0.140
No	23 (42.59%)	15 (28.85%)	
Yes	31 ( 57.41%)	37 ( 71.15%)	
History of arrhythmia			0.003
No	13 (20.07%)	27 (51.92%)	
Yes	41(79.93%)	25 (48.08%)	
Smoking habit			0.071
No	12 (22.22%)	8 (15.38%)	
Yes	38 (70.37%)	32 (61.54%)	
Past	4 (7.41%)	12 (23.08%)	
Drinking habit			0.002
No	14 (25.92%)	8 (15.38%)	
Yes	34 (62.96%)	22 (42.31%)	
Past	2 (3.70%)	15 (28.84%)	
Unknown	4 (7.42%)	7 (13.479%)	
History of coronary artery bypass graft			0.102
No	5 (9.26%)	1 (1.92%)	
Yes	49 (90.74%)	51 (98.08%)	
Treatment received			
Aspirin	54 (100%)	52(100%)	NS
Clopidogrel sulfate	54 (100%)	52(100%)	NS
ACEIs/ARBs			0.327
No	3 (5.56%)	1 (1.92%)	
Yes	51 (94.44%)	51 (98.08%)	
Beta-blockers			0.157
No	6 (11.11%)	2 (3.84%)	
Yes	48 (88.89%)	50 (96.16%)	
Calcium antagonists			0.327
No	3 (5.56%)	1 (1.92%)	
Yes	51 (94.44%)	51(98.08%)	
Nitrates			0.290
No	1 (1.85%)	3 (5.77%)	
Yes	53 (98.15%)	49 (94.23%)	
GPIIb/IIIa inhibitors			0.030
No	9 (16.67%)	2 (3.85%)	
Yes	45 (83.33%)	50 (96.15%)	

Data are presented as number (percent) or mean ± SD.

ACEIs = Angiotensin-converting enzyme inhibitors; ARBs = Angiotensin-receptor blockers; GPIIb/IIIa inhibitors = Glycoprotein IIb/IIIa inhibitors; NS = Not significant.

### Scr, cystatin C and eGFR 24 hours after treatment

Table 2 shows the levels of Scr and cystatin C as well as the eGFR 24 hours after treatment. After adjusting for age, body height, body weight, high-density lipoprotein, HBA1C, triglyceride, waist circumstance, small dense low-density lipoprotein, high molecular weight adiponectin, history of dyslipidemia, history of liver disease, history of cerebrovascular disorder, history of hypertension, history of cerebral hemorrhage, history of transient ischemic attacks, history of cerebrovascular disorders, history of arrhythmia, hmoking habit, hrinking habit, history of coronary artery bypass grafting, aspirin, clopidogrel sulfate, ACEIs/ARBs, beta-blockers, valcium antagonists, nitrates and GPIIb/IIIa inhibitors, the levels of Scr and cystatin C as well as the eGFR 24 hours after treatment were better than those of the control group (OR: 0.78, 95% CI: 0.54–0.82; OR: 0.66, 95% CI: 0.62–0.73; and OR: 1.2, 95% CI: 1.02–1.53, respectively).

**Table 2:** Scr, cystatin C and eGFR 24 hours after treatment

	Male	Female	Total
Scr at 24 h			
the control group	0	0	0
the TMZ groupl	0.72 (0.52, 0.85) 0.048	0.82 (0.43, 0.73) 0.036	0.78 (0.54, 0.82) 0.032
Cystatin C at 24 h			
the control group	0	0	0
the TMZ groupl	0.55 (0.0, 0.74) 0.041	0.69 (0.43, 0.77) 0.009	0.66 (0.62, 0.73) 0.022
eGRF at 24 h			
the control group	0	0	0
the TMZ groupl	1.25 (1.03, 1.92) 0.031	1.06 (1.02, 1.44) 0.000	1.2 (1.02, 1.53) 0.032

Results are presented as OR (95% CI) *P* value.

Adjust for: age, body height, body weight, high density lipoprotein, HBA1C, triglyceride, waist circumstances, small dense low density lipoprotein, high molecular weight adiponectin, history of dyslipidemia, history of liver disease, history of cerebrovascular disorder, history of hypertension, history of cerebral hemorrhage, history of transient ischemic attacks, history of cerebrovascular disorder, history of arrhythmia, hmoking habit, hrinking habit, history of coronary artery bypass graft, hspirin, hlopidogrel sulfate, ACEIs/ARBs, heta-blockers, valcium antagonists, nitrates, GPIIb/IIIa inhibitors.

### Scr, cystatin C and eGFR 48 hours after treatment

Table 3 shows the levels of Scr and cystatin C as well as the eGFR 48 hours after treatment. After adjusting for age, body height, body weight, high-density lipoprotein, HBA1C, triglyceride, waist circumstance, small dense low-density lipoprotein, high molecular weight adiponectin, history of dyslipidemia, history of liver disease, history of cerebrovascular disorders, history of hypertension, history of cerebral hemorrhage, history of transient ischemic attacks, history of cerebrovascular disorders, history of arrhythmia, smoking habit, drinking habit, history of coronary artery bypass grafting, aspirin, clopidogrel sulfate, ACEIs/ARBs, beta-blockers, calcium antagonists, nitrates, and GPIIb/IIIa inhibitors, the levels of Scr and cystatin C as well as the eGFR 48 hours after treatment were better than those of the control group (OR: 0.69, 95% CI: 0.52–0.73; OR: 0.76, 95% CI: 0.69–0.84; and OR: 1.5, 95% CI: 1.25–1.68, respectively).

**Table 3:** Scr, cystatin C and eGFR 48 hours after treatment

	Male	Female	Total
Scr at 48 h			
the control group	0	0	0
the TMZ groupl	0.62 (0.56, 0.75) 0.003	0.73 (0.55, 0.78) 0.016	0.69 (0.52, 0.73) 0.000
Cystatin C at 48 h			
the control group	0	0	0
the TMZ groupl	0.66 (0.53, 0.79) 0.014	0.83 (0.68, 0.92) 0.035	0.76 (0.69, 0.84) 0.002
eGRF at 48 h			
the control group	0	0	0
the TMZ groupl	1.35 (1.08, 1.63) 0.003	1.56 (1.42, 1.87) 0.000	1.5 (1.25, 1.68) 0.000

Results are presented as OR (95% CI) *P* value.

Adjust for: age, body height, body weight, high density lipoprotein, HBA1C, triglyceride, waist circumstances, small dense low density lipoprotein, high molecular weight adiponectin, history of dyslipidemia, history of liver disease, history of cerebrovascular disorder, history of hypertension, history of cerebral hemorrhage, history of transient ischemic attacks, history of cerebrovascular disorder, history of arrhythmia, hmoking habit, hrinking habit, history of coronary artery bypass graft, hspirin, hlopidogrel sulfate, ACEIs/ARBs, heta-blockers, valcium antagonists, nitrates, GPIIb/IIIa inhibitors.

### Scr, cystatin C and eGFR 72 hours after treatment

Table 4 shows the levels of Scr and cystatin C as well as the eGFR 72 hours after treatment. After adjusting for age, body height, body weight, high-density lipoprotein, HBA1C, triglyceride, waist circumstance, small dense low-density lipoprotein, high molecular weight adiponectin, history of dyslipidemia, history of liver disease, history of cerebrovascular disorders, history of hypertension, history of cerebral hemorrhage, history of transient ischemic attacks, history of cerebrovascular disorders, history of arrhythmia, smoking habit, drinking habit, history of coronary artery bypass grafting, aspirin, clopidogrel sulfate, ACEIs/ARBs, beta-blockers, calcium antagonists, nitrates, and GPIIb/IIIa inhibitors, the levels of Scr and cystatin C as well as the eGFR 72 hours after treatment were better than those of the control group (OR: 0.82, 95% CI: 0.77–0.91; OR: 0.85, 95% CI: 0.71–0.92; and OR: 1.67, 95% CI: 1.33–1.72, respectively).

**Table 4:** Scr, cystatin C and eGFR 72 hours after treatment

	Male	Female	Total
Scr at 72 h			
the control group	0	0	0
the TMZ groupl	0.76 (0.58, 0.79) 0.028	0.85 (0.67, 0.98) 0.001	0.82 (0.77, 0.91) 0.012
Cystatin C at 72 h			
the control group	0	0	0
the TMZ groupl	0.77 (0.58, 0.88) 0.000	0.89 (0.76, 0.94) 0.003	0.85 (0.71, 0.92) 0.000
eGRF at 72 h			
the control group	0	0	0
the TMZ groupl	1.32 (1.21, 1.55) 0.000	1.73 (1.36, 1.89) 0.033	1.67 (1.33, 1.72) 0.012

Results are presented as OR (95% CI) *P* value.

Adjust for: age, body height, body weight, high density lipoprotein, HBA1C, triglyceride, waist circumstances, small dense low density lipoprotein, high molecular weight adiponectin, history of dyslipidemia, history of liver disease, history of cerebrovascular disorder, history of hypertension, history of cerebral hemorrhage, history of transient ischemic attacks, history of cerebrovascular disorder, history of arrhythmia, hmoking habit, hrinking habit, history of coronary artery bypass graft, hspirin, hlopidogrel sulfate, ACEIs/ARBs, heta-blockers, valcium antagonists, nitrates, GPIIb/IIIa inhibitors.

### The incidence of CIN and MACE

Overall, CIN was diagnosed in 13.20% of all patients (14/106), and the prevalence of CIN was 9.26% (5/54) and 16.67% (9/52) between the TMZ group and the control group, respectively (*P* < 0.05). Furthermore, MACE occurred in 13.20% of all patients (14/106), and the prevalence of MACE was 7.41% (4/54) and 18.51% (10/52) between the TMZ group and the control group, respectively (*P* < 0.05).

### Sensitivity analysis

When stratified by gender, we found that the effects of trimetazidine on both men and women were similar and that TMZ could improve the renal function of patients (Tables 2–4).

## DISCUSSION

In our prospective study, we found that TMZ could reduce the levels of Scr and cystatin C after CAG/PCI and increase the eGFR after the operation. Meanwhile, after using TMZ, the incidence of CIN and MACE was significantly decreased. Therefore, our study indicated that TMZ can prevent CIN and MACE in diabetic patients with renal insufficiency who are undergoing CAG and/or PCI.

With the continuous development of coronary intervention, the incidence of CIN is increasing year by year [35–36]. Once CIN occurs, not only do the patient's hospitalization time and medical costs increase but their quality of life also significantly decreases, and CIN can even endanger the patient's life. At present, several studies [37–38] have noted that certain drugs can prevent CIN, but the clinical effects of these drugs are still controversial. In recent years, studies have found that TMZ can be used during CAG/PCI to prevent CIN in diabetic patients with renal insufficiency. However, this issue is still disputed.

We carried out a prospective study, and our findings also indicate that TMZ can prevent CIN and MACE in diabetic patients with renal insufficiency who are undergoing CAG and/or PCI. Our results are consistent with those of Onbasili AO and Shehata M. In 2007, Onbasili AO [39] carried out a study to assess the efficacy of TMZ in the prevention of CIN among patients with high serum creatinine levels undergoing CAG/PCI for the first time. A total of 82 patients were included in this study, Scr levels were measured before the procedure as well as 48 hours and 7 days after the procedure, and the results showed that Scr levels were significantly decreased in the TMZ group and that CIN developed in 2.5% (1/40) of patients in the TMZ group and in 16.6% (7/42) of patients in the control group (*p* < 0.05). In 2014, Shehata M [40] also conducted a prospective study to evaluate the effect of TMZ on the prevention of myocardial injury and CIN in diabetic patients with renal dysfunction who were undergoing elective PCI, and the research showed that the incidence of CIN was 12% in the TMZ group and 28% in the control group (*p* < 0.05) and that the level of troponin I was significantly decreased in the TMZ group (*p* < 0.001). Finally, Shehata M noted that the administration of TMZ to diabetic patients with renal insufficiency before elective PCI could decrease the incidence of CIN and myocardial injury.

At present, the exact pathogenesis of CIN has not been fully clarified. Some scholars believe that oxygen free radical injury plays an important role in the pathogenesis of CIN. A clinical study conducted by Bouzas [41] showed that lipid peroxidation was significantly increased and that the activities of superoxide dismutase and catalase were also significantly decreased in CIN patients. When a large dose of antioxidants (such as catalase) is administered, the impaired hemodynamics and renal function caused by contrast media were significantly improved, which indicates the pathogenic effects of oxygen free radicals in CIN. TMZ is an anti-ischemic metabolic agent that improves myocardial glucose utilization through the inhibition of fatty acid metabolism and is thus known as a fatty acid oxidation inhibitor. At present, underlying the prevention of CIN by TMZ is unclear. It may be related to the following aspects. First, animal experiments have noted that TMZ can reduce intracellular H+, Na^+^, Ca^2+^ overload, stabilize the mitochondrial membrane potential and decrease the production of oxygen free radicals, which elicit a variety of cardioprotective effects, such as anti-oxidative and anti-apoptotic effects [42]. Second, TMZ can improve the content of superoxide dismutase (SOD) in cells, inhibit the generation of oxygen free radicals, and increase the capacity of cells to remove oxygen free radicals [43]. Third, through cellular metabolism, TMZ can enhance mitochondrial activity, improve cell energy metabolism, reduce the release of oxygen free radicals, decrease the dissolution of and intimal injury to renal tubular epithelial cells, and relieve the toxicity of contrast agents on renal tubular epithelial cells [44].

### Limitations

Our study also has limitations that should be taken into consideration. First, 106 patients were included in this study. The sample size of this study is small, and it is possible that an increased sample size would result in opposite conclusions. Second, few have studied the use of TMZ in preventing CIN in diabetic patients with renal insufficiency. More clinical studies are still needed to confirm this conclusion. Third, studies using TMZ in the prevention of CIN are mainly conducted in Asia, Europe and North America. Therefore, the applicability of our study conclusions to patients in other regions requires more consideration.

## CONCLUSIONS

Despite several significant limitations, our study suggests that TMZ can reduce the incidence of CIN and MACE in diabetic patients with renal insufficiency who are undergoing CAG and/or PCI.
